# Supplementary figures and images for: The ELAV/Hu protein Found in neurons regulates cytoskeletal and ECM adhesion inputs for space-filling dendrite growth
Source: PLoS Genet. 2020 Dec 28;16(12):e1009235. doi: 10.1371/journal.pgen.1009235 (PMC7793258; doi:10.1371/journal.pgen.1009235)

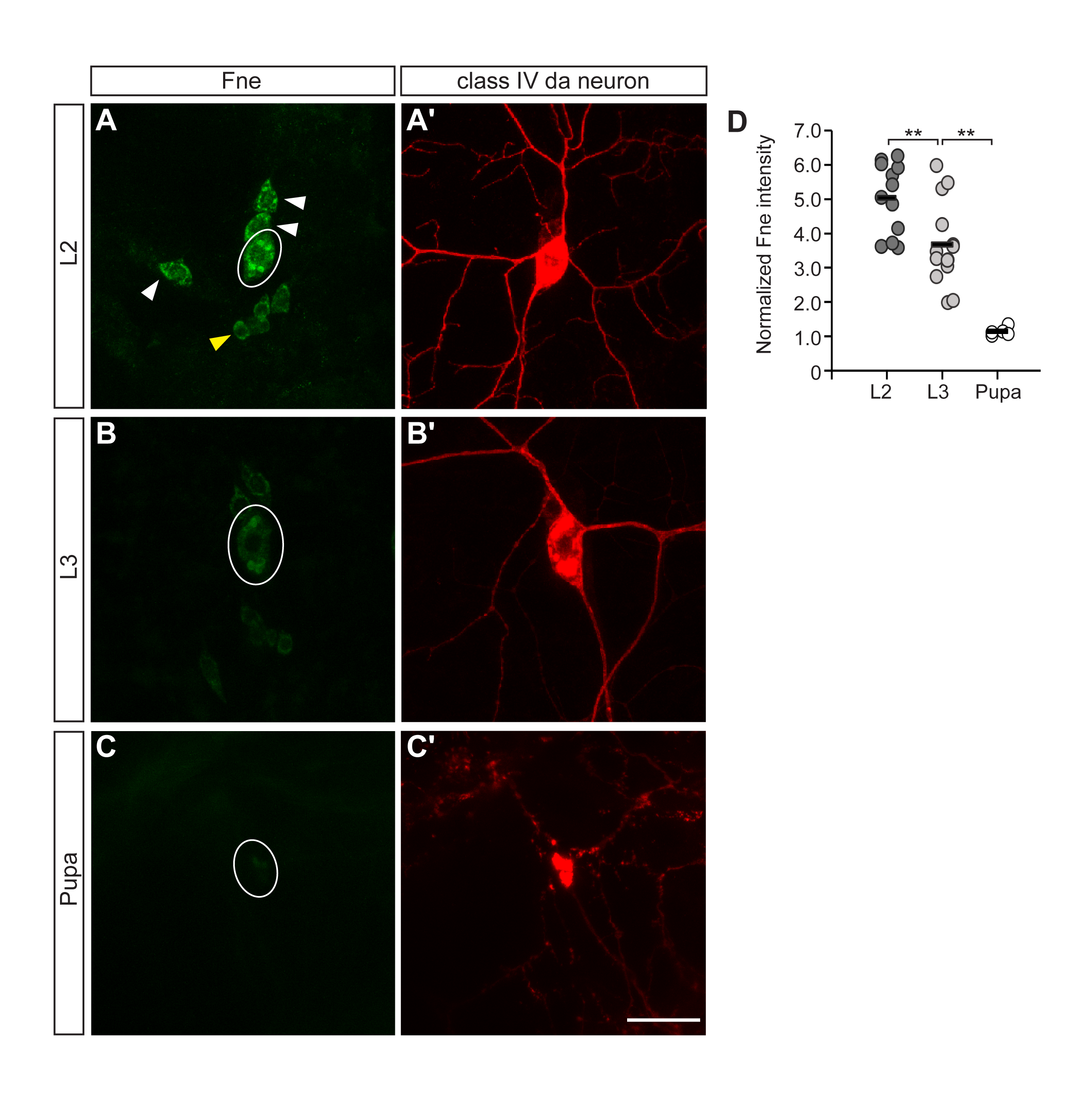

Supplement: S1 Fig — (A-C, A'-C') Confocal z-series projections of wild-type class IV da neurons labeled with anti-Fne (A-C) and anti-RFP to detect the CD4-Tom membrane marker (A'-C'). ppk-GAL4 was used to drive expression of UAS-CD4-tdTom. The class IV da neuron in each set of images is circled. Other classes of da neurons visible in (A) are indicated with white arrowheads, yellow arrowheads indicate external sensory (ES) neurons. (D) Quantification of Fne levels in class IV da neurons at L2 (n = 12 neurons), L3 (n = 14 neurons), and 10 h after puparium formation (APF; n = 5 neurons). Values are mean ± s.e.m.; **p<0.01 as determined by one-way ANOVA with Bonferroni-Holm post hoc test. Scale bar: 20 μm. (TIF) [file pgen.1009235.s001.tif]

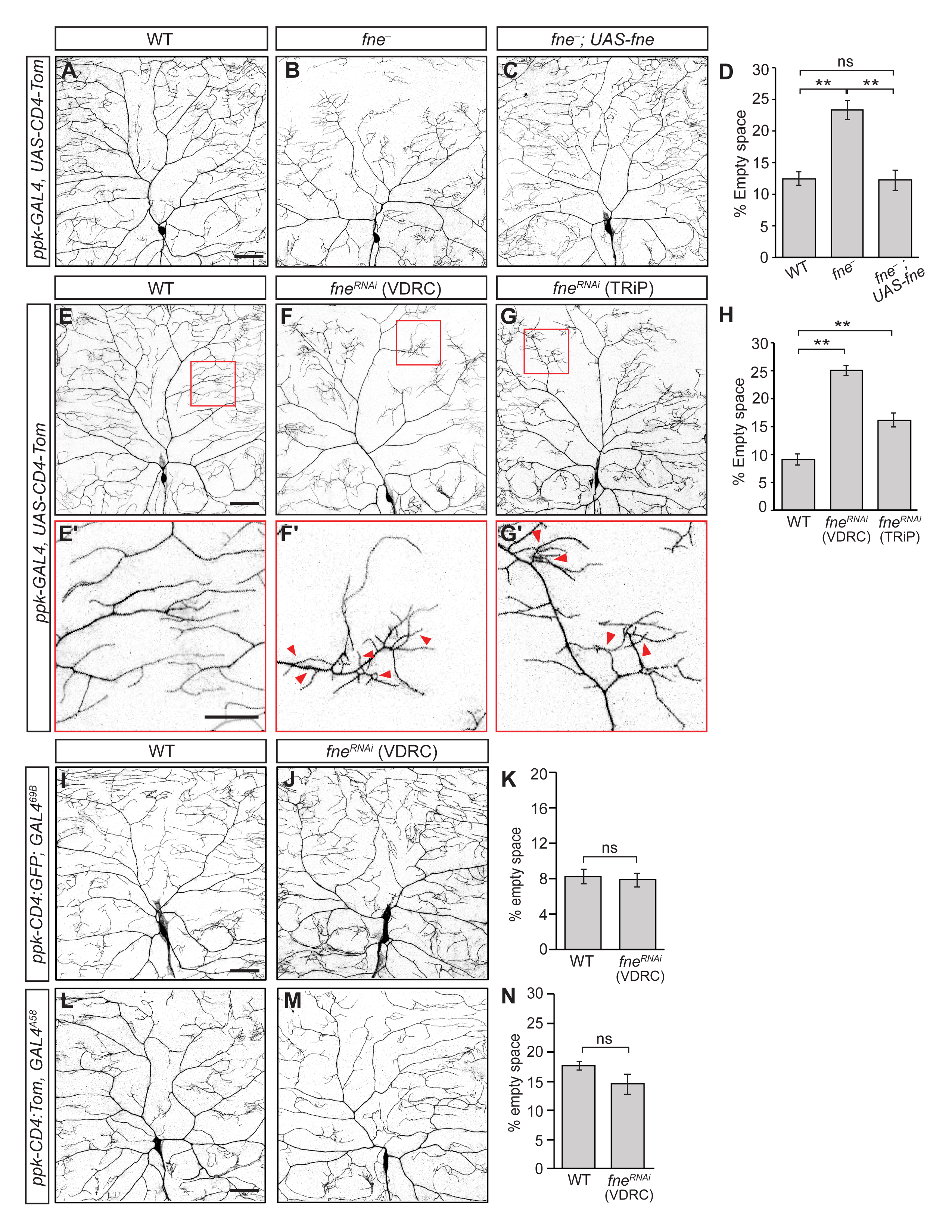

Supplement: S2 Fig — (A-C) Confocal z-series projections of wild-type (WT; A), fne−(B) and fne–; UAS-fne (C) neurons. ppk-GAL4 was used to express UAS-CD4-tdTom to mark the neurons and to drive UAS-fne expression. (D) Quantification of empty space in the arbor for WT (n = 11 neurons), fne−(n = 12 neurons), and fne–; UAS-fne (n = 10 neurons). (E-G) Confocal z-series projections of WT (E), fneRNAi (VDRC) (F) and fneRNAi (TRiP) (G) neurons. ppk-GAL4 was used to express UAS-CD4-tdTom to mark the neurons and to drive UAS-fneRNAi expression. Enlargements of the boxed areas in (E-G) are shown in E'-G'. (H) Quantification of empty space in the arbor for WT (n = 12 neurons), fneRNAi (VDRC) (n = 14 neurons), and fneRNAi (TRiP) (n = 12 neurons). (I, J) Confocal z-series projections of WT (I) and fneRNAi (VDRC) (J) neurons. Pan-epidermal expression of UAS-fneRNAiVDRC was driven using GAL469B and class IV da neurons were marked using ppk-CD4-tdGFP. (K) Quantification of empty space in the arbor for WT (n = 12 neurons) and fneRNAi (VDRC) (n = 13 neurons). (L, M) Confocal z-series projections of WT (L) and fneRNAi (VDRC) (M) neurons. Pan-epidermal expression of UAS-fneRNAiVDRC was driven using GAL458A and class IV da neurons were marked using ppk-CD-tdTom. (N) Quantification of empty space in the arbor for WT (n = 11 neurons) and fneRNAi (VDRC) (n = 10 neurons). Values are mean ± s.e.m.; **p<0.01 as determined by one-way ANOVA with Bonferroni-Holm post hoc test (D, H); ns = not significant as determined by two-tailed Student’s t-test (K, N). Scale bars: 50 μm (A, E, I, L), 20 μm (E'). (TIF) [file pgen.1009235.s002.tif]

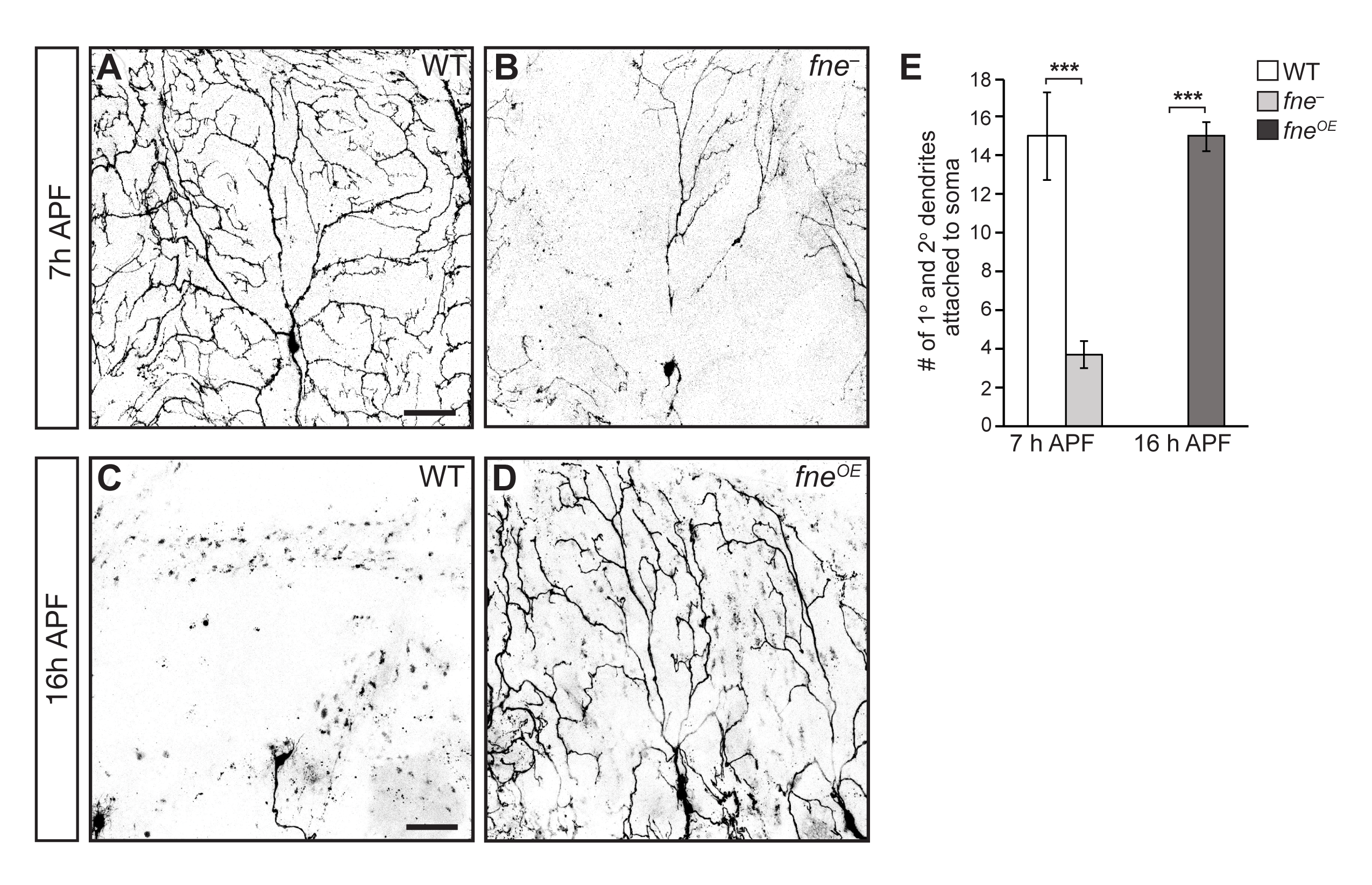

Supplement: S3 Fig — (A, B) Confocal z-series projections of wild-type (WT; A) and fne−(B) neurons 7 h APF. (C, D) Confocal z-series projections of WT (C) and fneOE (D) neurons at 16 h APF. ppk-GAL4 was used to drive expression of UAS-fne. For all genotypes, ppk-GAL4 was used to express UAS-CD4-tdGFP to mark the neurons. (E) Quantification of primary and secondary dendrites attached to the soma at 7 h AFP and 16 h APF: n = 8 neurons (WT, 7 h APF), 12 neurons (fne–, 7 h APF), 8 neurons (WT, 16 h APF), 6 neurons (fneOE, 16 h APF). Values are mean ± s.e.m.; ***p<0.001 as determined by two-tailed Student’s t-test. Scale bars: (A) 50 μm; (C) 100 μm. (TIF) [file pgen.1009235.s003.tif]

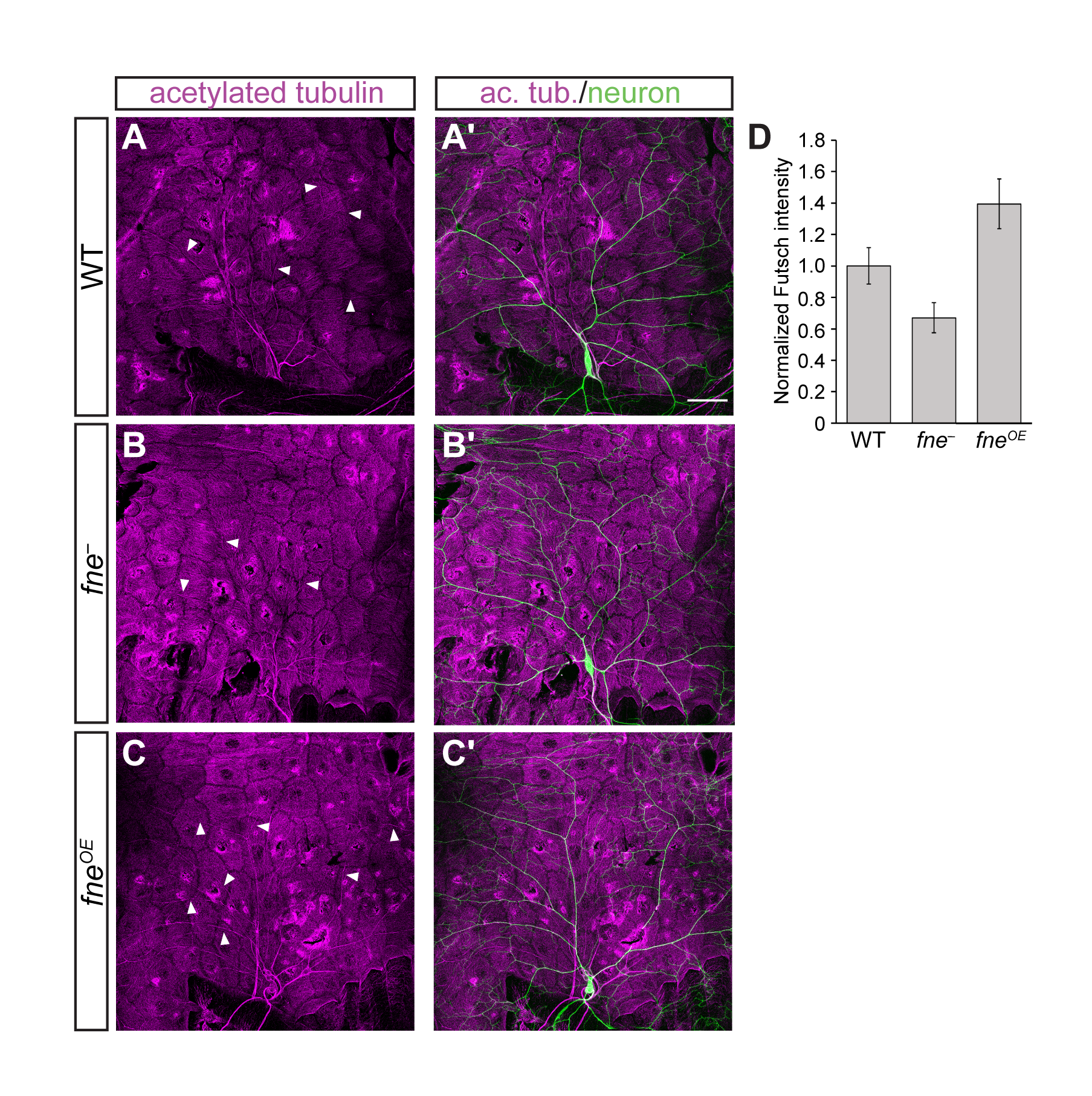

Supplement: S4 Fig — (A-C) Confocal z-series projections of wild-type (WT; A), fne−(B) and fneOE (C) neurons. ppk-GAL4 was used to express UAS-CD4-tdTom to mark the neurons and to express UAS-fne. Images show anti-acetylated tubulin (magenta, A-C, A'-C') to label stable microtubules and anti-RFP (green; A'-C') to detect CD4-Tom. Arrows indicate branches positive for acetylated tubulin. (D) Quantification of acetylated tubulin intensity normalized to the intensity of the membrane marker for WT (n = 9 neurons), fne−(n = 10 neurons), and fneOE (n = 13 neurons). Values are mean ± s.e.m. One-way ANOVA with Bonferroni-Holm post hoc test indicated no significant difference between the genotypes. Scale bar: 50 μm. (TIF) [file pgen.1009235.s004.tif]

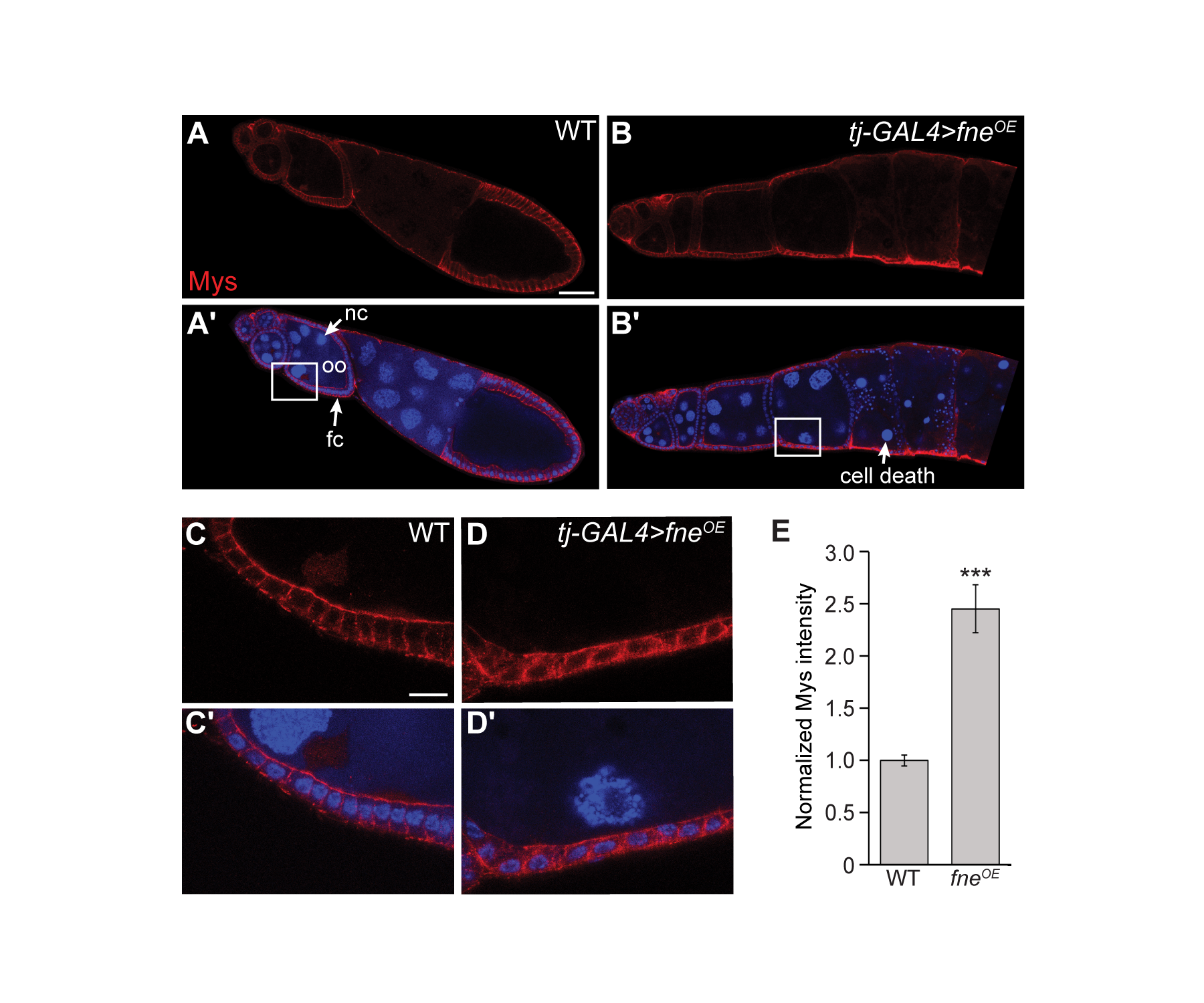

Supplement: S5 Fig — (A, A', B, B') Confocal z-series projections showing anti-Mys immunofluorescence (red) in wild-type follicle cells (WT; A. A') or follicle cells expressing UAS-fne driven by tj-GAL4 (B, B'). Follicle cells (fc), nurse cells (nc) and the oocyte (oo) are labeled. (C, C', D, D') Enlargements of the indicated areas in (A' and B'). Nuclei are labeled with DAPI (blue) in (A'-D'). (E) Quantification of anti-Mys immunofluorescence: n = 19 follicle cells (WT), 16 follicle cells (fneOE) from at least four egg chambers. Values are mean ± s.e.m.; ***p<0.001 as determined by two-tailed Student’s t-test. Scale bar: 10 μm. (TIF) [file pgen.1009235.s005.tif]

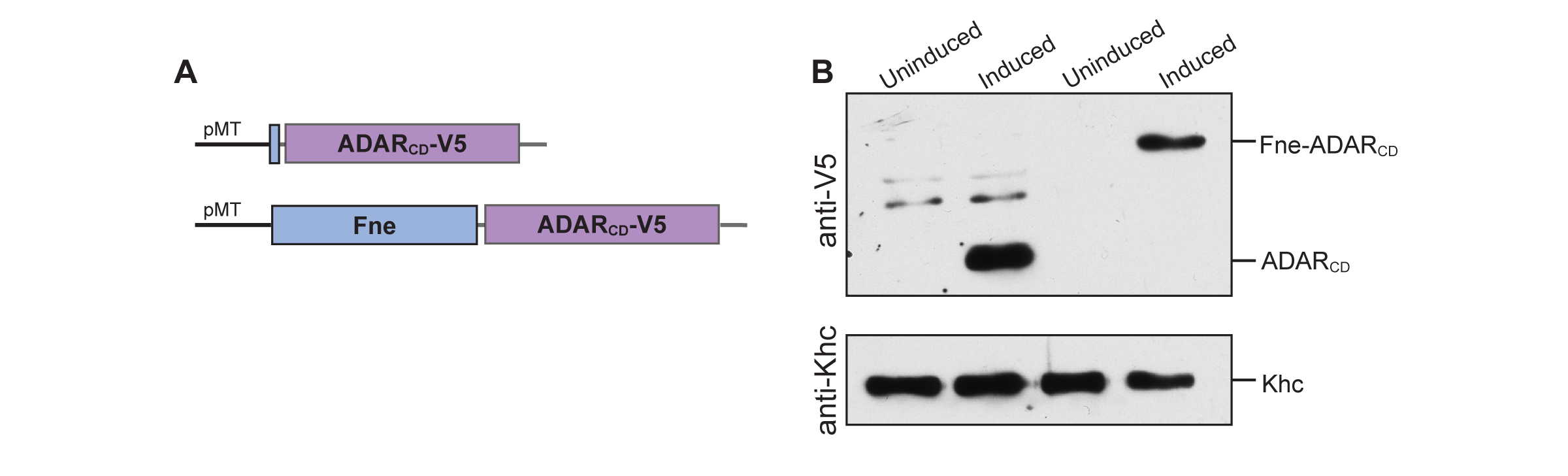

Supplement: S6 Fig — (A) Schematic of ADARCD and Fne-ADARCD constructs that were expressed in S2 cells. pMT denotes the metallothionine promoter and V5 denotes the V5 epitope tag. (B) Western blot of protein isolated from stable cell lines before and after induction with CuSO4. Anti-V5 antibody was used to detect ADARCD and Fne-ADARCD, anti-Khc antibody was used to detect Kinesin heavy chain (Khc) as a loading control. (TIF) [file pgen.1009235.s006.tif]

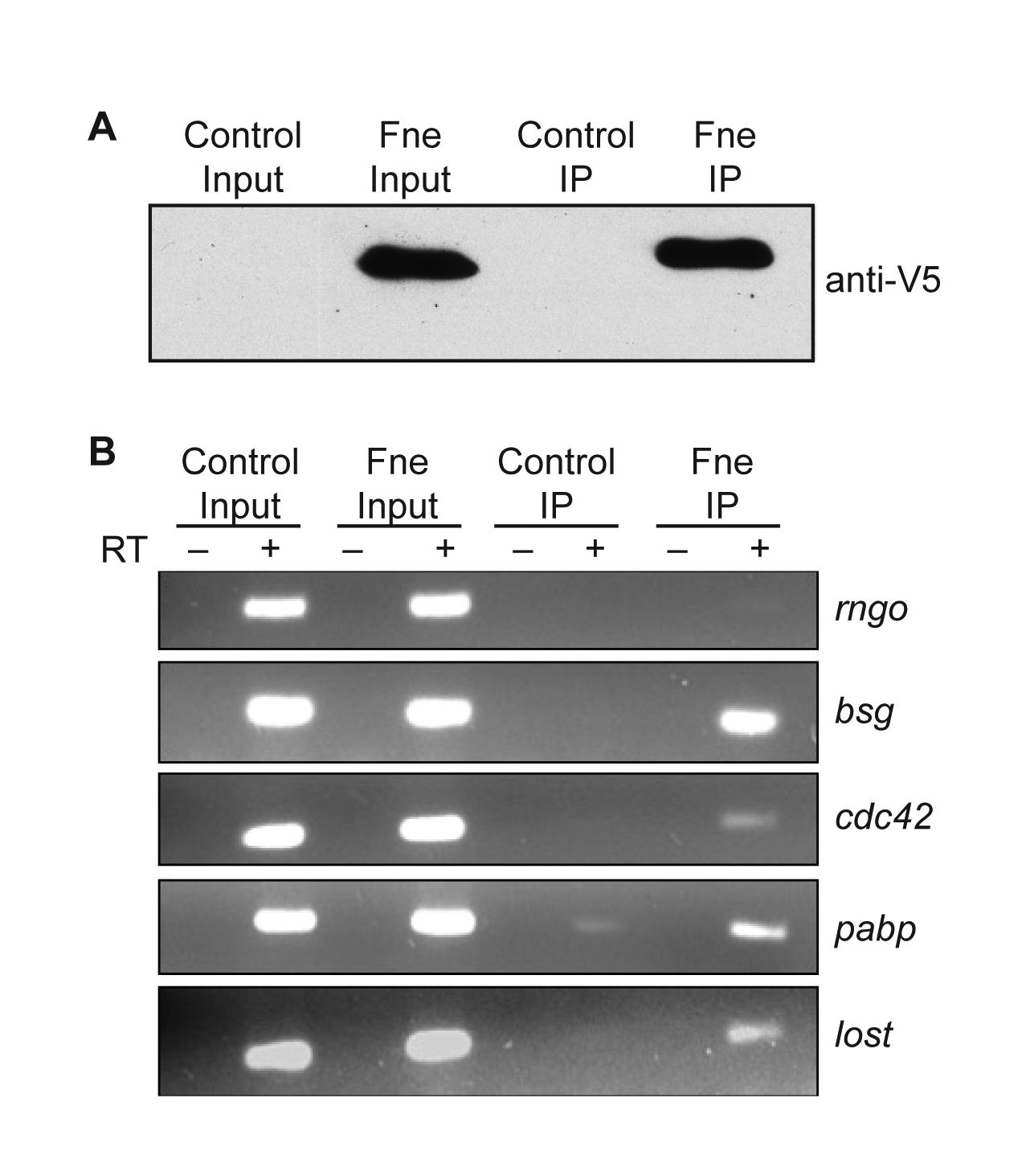

Supplement: S7 Fig — (A) Anti-V5 immunoprecipitation of cell lysates obtained from stable cell lines with or without induction with CuSO4. Anti-V5 antibody was used to detect Fne-V5. (B) Whole cell extract and immunoprecipitates were analyzed by RT-PCR to detect bsg, cdc42, pabp and lost. rngo was used as a negative control, as it is abundant in Drosophila S2 cells, but was not identified as a target of Fne by TRIBE and does not show overlap with the enriched GO terms for the set of target RNAs. (TIF) [file pgen.1009235.s007.tif]

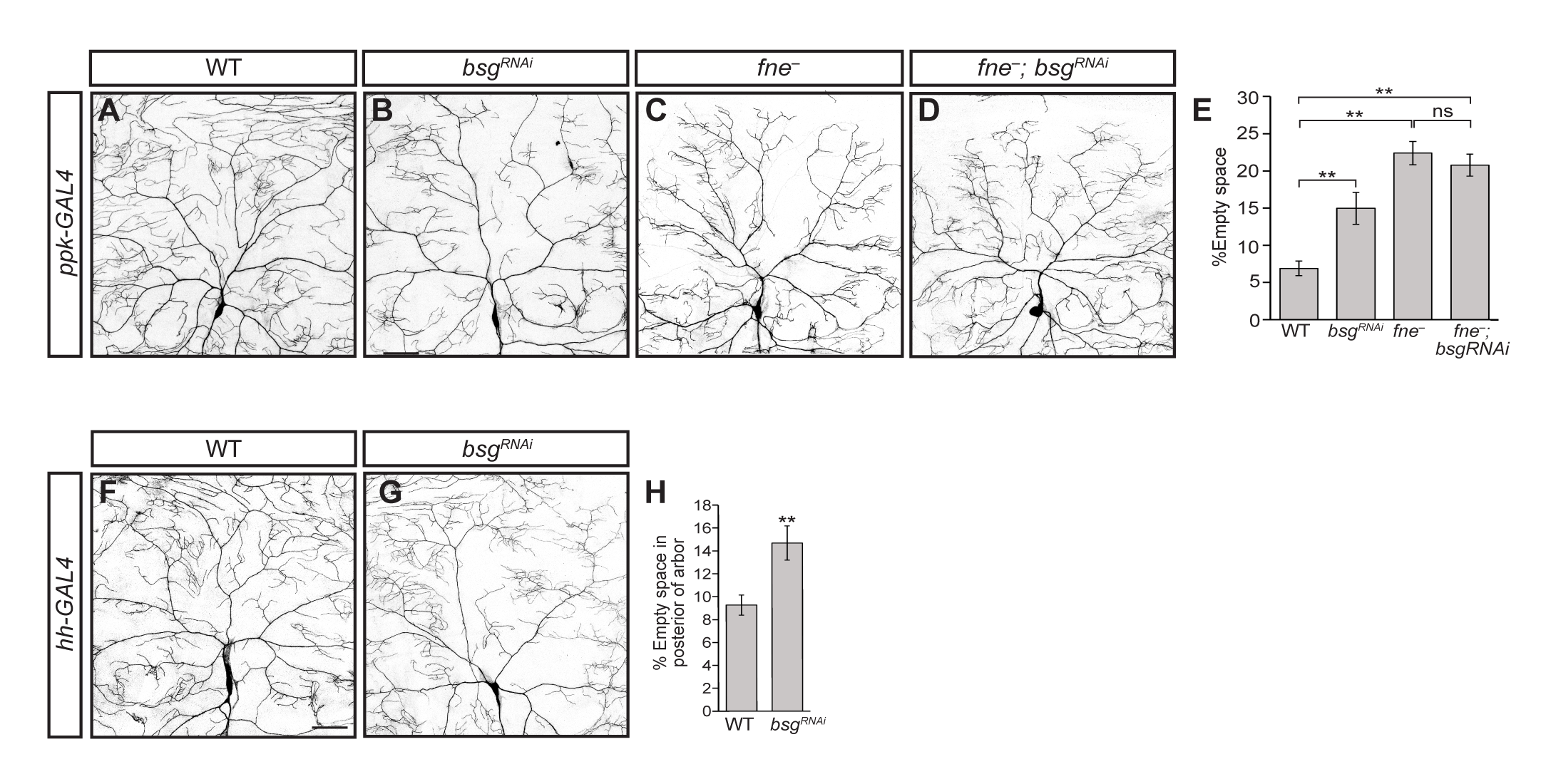

Supplement: S8 Fig — (A–D) Confocal z-series projections of wild-type (WT; A), bsgRNAi (B), fne−(C) and fne–; bsgRNAi (D) neurons. ppk-GAL4 was used to drive expression of UAS-CD4-tdTom to mark the neurons and to drive expression of UAS-bsgRNAi (BDSC 52110). (E) Quantification of empty space in the arbor for WT (n = 9 neurons), bsgRNAi (n = 7 neurons), fne−(n = 6 neurons), and fne–; UAS-bsgRNAi (n = 7 neurons). (F, G) Confocal z-series projection of neurons from a WT (F) larva and a larva expressing UAS-bsgRNAi (BDSC 52110) in the posterior portion of the epidermis driven by hh-GAL4 (G). Neurons were marked with ppk-CD4-tdGFP. (H) Quantification of empty space in the posterior third of the arbor for WT (n = 17 neurons) and bsgRNAi (n = 16 neurons) in (F, G). Values are mean ± s.e.m.; **p<0.01 as determined by one-way ANOVA with Bonferroni-Holm post hoc test; ns = not significant. Scale bars: 50 μm. (TIF) [file pgen.1009235.s008.tif]

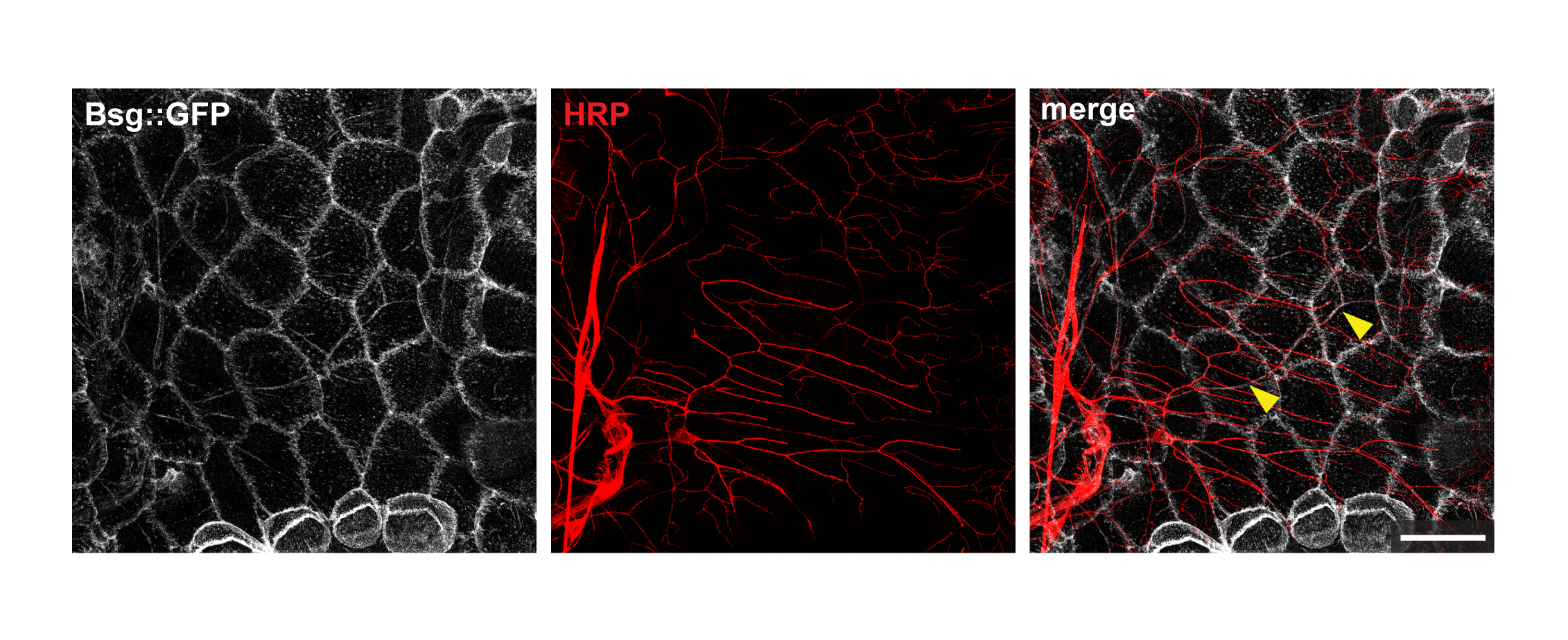

Supplement: S9 Fig — Anti-HRP immunofluorescence (red) of a late L3 larva expressing Bsg::GFP (white). Anti-HRP immunostaining was performed prior to tissue permeabilization and therefore detects unenclosed dendrite segments (see Materials and methods). Bsg::GFP localizes to areas of dendrite enclosure, as marked by the absence of HRP signal (yellow arrows in merged image). Note that HRP detects other unenclosed neurons in the field. Scale bar: 50 μm. (TIF) [file pgen.1009235.s009.tif]
