# Supplementary material for: The ELAV/Hu protein Found in neurons regulates cytoskeletal and ECM adhesion inputs for space-filling dendrite growth
Source: PLoS Genet. 2020 Dec 28;16(12):e1009235. doi: 10.1371/journal.pgen.1009235 (PMC7793258; doi:10.1371/journal.pgen.1009235)
Supplement: Attachment — Submitted filename: Response to Reviewers_final.docx [file pgen.1009235.s014.docx]

We are happy that the reviewers found our work interesting and we thank them for their helpful comments and suggestions. We have addressed all of the criticisms to the best of our ability given the constraints of the pandemic. To do so, we have performed 8 additional experiments (Fig. 4D-G), Fig. S1 (B-D), Fig. S2 (3 expts), Fig. S4, Fig. S8 (A-E), (Fig. S9), added data (Fig. 6G), reanalyzed data (Fig. 5) and made numerous modifications to the text. All of the changes are detailed below. We believe that these substantial revisions have greatly improved the manuscript and hope that the reviewers will agree.

**Reviewer #1**

*Major concerns:
1. The fne mutant displays quite a few interesting stage-dependent dendritic phenotypes, such as extra proximal and shorter branches in the L2 stage, raising the possibility that Fne’s function or expression is temporally (and spatially) regulated. There is only one image showing Fne expression in class IV da neurons (Fig. S1), and the specific larval stage for image was not described. What are Fne expressions in different larval and pupal stages? What are the cells/neurons that are also Fne-positive in Fig S1 (in addition to the class IV da neuron)?*

- We performed additional immunofluorescence experiments and have added images showing that Fne levels appear to decrease as the animal progresses from L2 to the pupal stage and circled the Fne positive cells (Fig S1). The other cells, now indicated by yellow arrowheads, are external sensory neurons.

*2. If Fne is also expressed in other classes of da neurons, do they show dendrite phenotypes in fne mutants? In particular the class I da neuron which is not space-filling but microtubule-dominant in dendrites, a contrast to the class IV da neurons****.***

- We have not analyzed *fne* function in other classes of da neurons. We agree that this would be an interesting direction for future work but are limited in what we can do at this time.

*3. The cytoplasmic distribution of Fne in Fig S1 does not correlate with local action of Fne to regulate microtubules and Bsg in dendrites. Have fluorescence protein-Fne fusions been examined in the dendrites to examine the protein localization?*

- Unfortunately, there are currently no Fne-FP lines available to answer this question. We do not know whether Fne works locally (but below the limit of detection by the antibody) or whether, as an RNA-binding protein, its effects are far-reaching and are not limited by its localization. We have been careful not to claim that Fne is acting locally in the dendrites.

*4. Microtubule phenotype in fne mutants is suggested only by the reduction of Futsch immunostaining. Other microtubule makers should be examined to suggest the reduction in stable microtubules.*

- We examined a second marker, acetylated tubulin, in *fne* mutant and overexpressing neurons. Unlike Futsch, this marker is discontinuous in da neuron dendrites and, more problematically, is not specific to neurons and high levels in epidermal cells make quantification of neuronal levels difficult. We have had similar difficulties with Jupiter. Our results do show a similar trend to Futsch, with decreased levels of stable microtubules in *fne* mutant neurons and increased levels in *fne* overexpressing neurons. The values do not reach significance as determined by ANOVA with Bonferroni-Holm *post hoc* test, however, likely due to interference from the signal in the epidermal cells as well as the discontinuous signal in the dendrites. The data have been added to the manuscript as Fig. S5.

*5. The role of Fne in the integrin pathway for dendrite-ECM interaction is very indirect, as suggested by the increases in branch enclosure and cross-over. One would expect to perform genetic interaction with integrin subunits to suggest an involvement. The overexpression of Fne to disturb Mys localization in follicle epithelial cells is not supportive, either. What is the localization of Myc in loss of fne mutants?*

- We have now tested genetic interaction of *fne* with integrins as recommended by the reviewer and we show that overexpression of integrin (βPS and PS1 subunits) rescues the crossing over defects observed in *fne* mutant neurons. The new data have been added to Fig. 4.
- Because Mys is highly expressed in the epidermis as well as in the neuron and the two are closely apposed, we cannot distinguish Mys in dendrites from epidermal Mys using confocal microscopy. We previously had limited success with super resolution microscopy but due to the COVID pandemic, we no longer have access to the necessary microscope. The follicle cell data will be moved to the supplement, as suggested by Reviewer 2 (Fig. S6).

*6. The authors describe that “Epidermal knockdown of bsg using hh-GAL4 led to similar defects” to ppk-GAL4 knockdown. This is not clear! The hh-GAL4 knockdown only affect the posterior portion of the segment. Does the dendrite phenotype is also present in the anterior part?*

- We apologize for the confusion. The phenotype is only manifest in the posterior region of the arbor. We have modified the text on p.13 to be more clear: “Epidermal knockdown of *bsg* using *hh-GAL4* led to similar defects in the posterior portion of the arbor, which underlies the epidermal expression domain of *hh-GAL4*” and have also cited Poe et al. (2017) PNAS *114*, E8062-E8071 who documented the expression domain of *hh-GAL4* by using it to drive *UAS-EGFP*.

*7. Since Bsg also regulates microtubule stability, what are the terminal branch dynamics in bsg mutants? Does it phenocopy fne mutant in branch dynamics?*

- We performed live imaging of *bsg RNAi* neurons and found that terminal branch dynamics were not affected. This suggests that the decrease in Futsch alone is not enough to cause the observed branch instability observed in *fne* mutant neurons and that it may instead result from loss of contact between terminal dendrites and the ECM (Fig. 4) or possibly to an effect on actin dynamics that we could not detect using LifeAct. We have edited the Discussion to reflect this result. We note that other studies have shown that decreased Futsch does affect branch initiation or elimination (Jenkins et al. [2017] *130*, 4120–4131), and conversely, decreased branch stability is not necessarily correlate with changes in Futsch (Stewart et al. [2012] J. Neurosci. *32*, 11631-42), indicating that the relationship between Futsch and terminal branch stability is complex.

Minor comments

*1. The usage of “CD4:GFP” and “CD4:TOM” are not conventional! Please check the flybase and correct them!*

- We have modified the nomenclature to be consistent with other papers in the field.

*2. Fig 1I showing the Sholl analysis is not very clear! It could be separated into two sub-figures for L2 and L3.*

- Thank you for pointing this out. We have separated the data to make it more clear (Fig. 1I and 1J).

*3. In Fig 1L, the number of crossovers per 1000um were scored. It is not clear what are does the “1000um” stand for ( not described in Fig legend!)? One way is to score crossover per branch!*

- We have clarified that the number of crossovers is normalized to branch length in both the legend and the methods. A similar quantification was performed in Han et al. (2012) Neuron *73*, 64-78 and Kim et al. (2012) Neuron *73*, 79-91. We obtained a similar result for crossovers/1000 µm in wild-type neurons as was shown in these two previous studies.

*4. What are the terminal phenotypes in pruning? Do dendrites regrew in fne mutants after premature pruning?*

- We agree these are interesting questions, but we have no experience with the vastly different adult PNS and have therefore chosen to focus our detailed analysis primarily on the larval phenotypes.

*5. There are two Fig 5B’s in Fig 5!*

- We have corrected this error.

**Reviewer #2:**

*1) The authors performed a Scholl analysis to determine the critical radius, a measure integrating local dendrite density over the surface of the arbor. On page 10, they state: “Arbors of fneOE neurons showed (…) a greater critical radius than either wild-type or fne– arbors ». There is one outlier data point in the set used to calculate the critical radius of OE (Fig 1-J), which, by itself, could account for the difference in critical radius between fne- and fneOE L3. Morphological differences of the dendritic arbors are fairly apparent (Fig 1. L2 and L3), raising the possibility that the Scholl analysis may not be the most appropriate. Perhaps a branch order analysis (Strahler) would provide better quantification of the internal organization/complexity of the arbor?*

- We agree that while both *fne* mutant and overexpressing neurons show increased empty space, qualitative differences exist between *fne* mutant and overexpressing arbors. However we have not found a suitable way to quantify this. Strahler analysis of these arbors proved to be difficult and unreliable due to the crossing over seen in *fne* mutant neurons and the bunching seen in *fne^OE^* neurons, both of which make it difficult to track branches definitively back to their origin. We also believe that because of the bunching of terminal branches seen in *fne^OE^* neurons, Sholl analysis does not capture the full phenotypic defects of these arbors. Thus, we are somewhat limited in the quantitative analysis of *fne* mutant and overexpressing arbors and have analyzed them to the best of our abilities. We think that readers will be able to appreciate the qualitative defects in these arbors.

*2) The fne OE allele was used here in several experiments, compared with fne- and WT animals, in most cases yielding outcomes that are easy to interpret in genetic terms. Such is not the case however for the measure of % of empty space, which doubles in fne- and fneOE larvae compared to WT (Fig 1L, ***). The authors should provide their interpretation for the identity of the phenotypes of fne loss of function and over-expression in this experiment.*

- We have added our interpretation of the loss of coverage for both *fne* mutant and overexpressing neurons to the Discussion (p.15).

*3) A percentage, whose definition is unclear, was used as a quantification unit for the crossovers in Figure 4C. Why not use the same unit as in Fig 1L, where crossovers are reported by dendrite length? Also, it should be specified if the crossovers were measured on terminal non-enclosed dendrites or on all dendrites. The larval stage (presumably L3) should be specified and the arrow heads in Fig 4A need to be defined.*

- The percentage in Fig. 4C is the number of crossovers that occur in regions where dendrites are enclosed divided by the total number of crossovers (i.e., the proportion of all crossovers that occur in regions of enclosure). This metric differs from the quantification in Fig. 1L where we simply quantify the frequency of crossovers (the number of crossovers per dendrite length). In Fig. 4C, we show that the crossovers are more likely to occur in enclosed regions of the arbor.
- We have indicated the larval stage (L3) and explained the arrowheads in the figure legend.

*4) Dendrite-ECM contacts depend upon integrins, leading the authors to test whether fne might be regulating the expression of integrins. To circumvent technical difficulties, the experiment was performed in oocytes rather than neurons. Ectopic expression of fne in follicle cells (tj-gal4 driver) led to an increased level of the predominant beta-integrin subunit, Mys (almost 2.5 fold increase, p<0.001). In these conditions, the distribution of Mys in follicle cells became less peripheral, and the cells became flatter. The authors conclude that fne is part of a pathway that up-regulates Mys expression and distribution.*

*Question:*

*This experiment is obviously of interest, but its relevance to C4da development is unclear. It would only be an extrapolation of the conclusions gathered in the follicle cells to conclude that Mys is promoting space-filling dendrite growth of the C4da neurons. Should this experiment be moved to the supplemental data?*

- As suggested, we have moved the follicle cell data to the supplement (Fig. S5).

*5) I understand that the authors want to make the assumption that “many fne targets are not going to be neuron specific” (page 10), since the biological material that they use (S2 cells) is not neuronal. But there is no evidence for this. Along the same line of reasoning, the population of RNA identified with TRIBE may or may not represent the targets of FNE. This is not only because of the biological material used, but also because of the low sensitivity of the method (partial editing, see: RNA 24: 173). A third issue affecting the output of TRIBE is the statistical analysis: In the Section “TRIBE assay”, it is stated that « The same thresholds used by Rahman et al. were applied here, namely a minimum of 20 reads and a minimum editing frequency of 10% ». The Rahman et al reference is about an improved protocol (hyperTRIBE). These thresholds are too conservative for TRIBE according to the Rosbash lab, who developed the methods (ibid). Therefore the TRIBE experiment must be identifying only a (small) subset of Fne targets. As a consequence, the GO analysis presented in the manuscript and the discussion about expected/predicted Fne targets that were not identified by TRIBE (specifically Mys, page 15) are not strong and bury the essential information/conclusions.*

- We believe that S2 cells are a reasonable source for this experiment because the cell biological processes affected by Fne – cytoskeletal organization (Fig. 3) and cell-ECM adhesion (Fig. 4) –­ are not neuron-specific. We completely agree that some targets will not be identified, either because of the sensitivity of the TRIBE method (we performed the experiment long before HyperTRIBE was published) or because some targets may not be expressed in S2 cells and we have acknowledged these limitations. We have modified the text in the Results section to explicitly state that TRIBE can only identify a subset of Fne targets due to the limited sensitivity of the method. In the Discussion, we indicate that it is not surprising that expected targets were missed.
- We have now reanalyzed the data using a 5% threshold and have updated Fig. 5 to reflect this. Using a lower threshold led to the identification of more targets, but did not ultimately change the GO term enrichment or any of the other parameters shown in Fig. 5. We have provided more information regarding our GO analysis in the "TRIBE Analysis" methods section (page 24).
- We have removed the term “unbiased” from statements about TRIBE results to recognize the fact that only a subset of Fne targets are identified in our analysis. Although we were using “unbiased” to reflect that our analysis was not directed towards identifying particular Fne targets, we understand the confusion this term causes.
- We have added a Data availability statement: The TRIBE raw datasets will be publicly at NCBI GEO.

*6) Bsg expression was assessed using one GFP reporter. GFP fluorescence is seen at the periphery of epidermal cells (main signal) and along sections of the dendritic tracks (Fig 6BC, Figure 7A). Most of the Bsg-GFP fluorescence is lost when hh-GAL4 drives a Bsg RNAi (Fig 6BC). The authors conclude that Bsg is expressed in epidermal and neuronal cells. I am intrigued by the data shown in Figure 7 A-A’ where it seems that GFP expression (Bsg+) goes beyond the soma of a CD4da neuron, in a territory that seems neither epidermal nor dendritic.*

- This is likely Bsg in the somas of other neurons not detected by the neuronal marker, which is expressed specifically in class IV da neurons.

*Issues:*

*The determination of the pattern of expression of Bsg (in Fig 6) raises questions about some crucial parameters regarding the reagents that were used for that purpose.*

*What is the exact cell/tissue specificity of hh-Gal4 expression in C4da neurons? (stated to be epidermal, but its neuronal expression in the brain has been demonstrated)*

Is there any leakiness of the RNAi line in C4da neurons?

- Poe et al. (2017) PNAS *114*, E8062-E8071 showed that *hh-GAL4* is specifically expressed in the epidermis, without any leakiness in class IV da neurons.

*How specifically is the Bsg-GFP reporter expressed in C4da neurons?*

- We used a GFP-trap in the endogenous *bsg* gene, so Bsg::GFP is an endogenously expressed reporter and is consequently detected where Bsg is normally expressed.

*There is a need to provide/cite experimental or scholarly evidence to answer these questions. This is especially important in view of the fact that (1) A perineurial (and subperineurial) glial layer ensheathes the proximal dendrites of da neurons and (2) recently, Hunter et al have found that a Bsg::GFP reporter and integrin colocalize in the perineurial glia of Drosophila larval peripheral nerves (J. Neurosci.40: 3360).*

- Glia wrap the soma and portions of proximal dendrites of class IV da neurons (Han et al. [2011] PNAS *108*, 9673-9678), whereas the Bsg tracks extend much farther out into the arbor. Furthermore, the *ppk-GAL4* and *hh-GAL4* drivers are specific to the neuron and epidermis, respectively. Thus, although Bsg could be expressed in the glia surrounding the soma, we believe this source of Bsg is not the target of Fne nor is it relevant to the function of Bsg regulating the stable MT population in the arbor.

*Questions:*

*Assuming that the Bsg:GFP fluorescence in the dendrites can be reproduced with other reporters, I am intrigued by its discontinuity along the tracks. Tom+ GFP- sections of dendrites are visible (Fig 7A). Would it be possible that there is any connection with dendritic enclosure?*

- We show that Bsg localizes to regions of dendrite enclosure (new Fig. S9). However, unlike *fne* mutants, loss of *bsg* does not result in a change in dendrite crossing events. This indicates that the effect on enclosure observed in *fne* mutants is not a consequence of reduced Bsg levels, and suggests that while Bsg positive dendrite segments are enclosed by epidermal cells, Bsg is not an active participant in the enclosure process. We have added explanations on pp. 14 and 16.

*7) The GFP fluorescence of the Bsg::GFP reporter along the dendritic tracks and in the epidermal cells is quasi abolished in fne- mutants (Fig 7B’), suggesting that fne stimulates Bsg expression. The authors propose a plausible interpretation for the extinction of epidermal Bsg expression by neuronal fne: in the absence of neuronal fne, neuronal Bsg expression is diminished, and does not recruit epidermal Bsg for homophilic interactions, hence the neuronal and ectodermal Bsg depletion in fne mutants. To support their model, the authors make the valid prediction that under the hypothesis that fne stimulates Bsg expression, defects in fne OE will be corrected by Bsg RNAi.*

*However, the use of % of empty space in the dendritic arbor to evaluate their prediction is of concern. They showed earlier in this manuscript that compared to WT, the % of empty space in* *dendritic arbors roughly doubles in fneOE, but in fne- mutants as well (Figure 1H). There are clearly many ways to increase the % of empty space in the dendritic arbor, and in consequence the interpretation of the experiment shown in Fig 7 is not straightforward.*

*Furthermore, there is about 22% empty space in fneOE, about 16% empty space in Bsg RNAi, and 12% in WT (Figure 7), leaving very little margin to detect convincing suppression.*

*A suitable variable to assess the suppression by Bsg RNAi of fneOE defects on the dendritic arbor should be affected in opposite ways in fne- and fneOE. This is the case for the normalized Futsch intensity that the authors examined. They observe a very moderate suppression of the fne OE defects in Futsch intensity. A tight control of the experimental conditions would be necessary to bring clear answers.*

- Respectfully, we are unclear on what the reviewer is requesting. Although *fne* mutant and overexpressing neurons both show an increase in empty space, *bsg* RNAi partially suppressed the empty space defect in *fne^OE^* mutant neurons (Fig. 7) but did not restore coverage in *fne^–^* neurons (p. 13, new data added in Fig. S8). This experiment shows that although the rescue by *bsg* RNA is only partial (but still significant with the proper tests as requested by Reviewer 3), it is specific to *fne^OE^*, despite the similarity between *fne^OE^* and *fne* mutant phenotypes, and thus consistent with the idea that *fne* positively regulates *bsg*. We are also careful not to claim that *bsg* is the only target of Fne.
- Our Futsch analysis does detect robust and opposing effects by *bsg* RNAi and *fne^OE^* (Fig. 7L) with *bsg* RNAi clearly reproducing the *fne* mutant phenotype (Fig. 3D). The magnitude of the changes in Futsch are consistent with those observed in other studies on Futsch in class IV da neurons (Jenkins et al. [2017] *130*, 4120–4131, Hu et al. [2020] J. Neurosci. *40*, 1819-1833). We agree that suppression of the *fne^OE^* effect on Futsch intensity is modest and we have clarified this in the Results ("neuronal knockdown of *bsg* modestly suppressed the elevated dendritic Futsch levels caused by *fne^OE^*"). Additionally, in the abstract we state: "the cytoskeletal defects contributing to branch instability in *fne* mutant neurons are due in part to decreased Basigin expression the statement" and we make a similar statement in the Introduction.
- We have also added the statement "Our TRIBE results suggest that the morphological	defects seen in *fne* mutant neurons are the result of misregulation of many target	transcripts" to the Introduction.

*MISCELLANEOUS GENERAL COMMENTS:*

*Quantitative and statistical results should be reported in the Results section. For instance, rather than “excess of terminal branches”, specify “excess of terminal branches (two fold, p<00.5)”. The information is in the Figures/legends but the critical information should be in the text of the manuscript.*

- We have added numerical data and p values to the text in the Results section.

*Please indicate the complete genetic composition of the tissues shown in Figures 6, 7, 5S, with the specific names of the Bsg::GFP and RNAi lines (on the figure or its legend). The specifics of all the stocks used should be listed.*

- We have added this information to the figures and/or legends.

In Figure 6B-C, the two scale bars do not appear consistent and the yellow rectangle used to mark the magnified area is not covering the relevant field.

- We have corrected this error.

*I am curious to know if animals of only one sex were used in the experiments (at least the third instar larvae which are the main material used for this analysis)? Sexing animals is a good precaution to decrease internal variability in data sets.*

- For crosses involving *fne* mutants, only males were used because *fne* is on the X-chromosome. For all other genotypes, males and females were both used. In our experiments not presented here, we have consistently found no difference in class IV da neuron dendrite morphology between male and female larvae.

*For Fig 6, panel D-I and Fig 7 H-L: The Methods section mentions: “Crosses with UAS-bsgRNAi were performed at 29°C to increase GAL4/UAS efficiency, and all other crosses were performed at 25°”. I assume that the authors meant “crosses involving UAS-bsgRNAi and their controls were performed at 29°C to increase GAL4/UAS efficiency, and all other experiments were performed at 25°”.*

- Thank you for pointing this out. It has been corrected.

*Reference 45 is mis-cited as ref 46 at the bottom of page 11.*

- We have corrected this mistake.

Finally, the term “upstream regulator” is used several times in the text to qualify fne. This is a little awkward, since fne is an RNA binding protein. Usually, this term is used for molecules that associate in the upstream region of a gene.

- We used the term upstream regulator to indicate that Fne regulates targets that lie upstream in a pathway. We appreciate that this could cause confusion and have therefore changed the wording to “Fne acts to modulate both cytoskeletal composition and dendrite-ECM adhesion.”

**Reviewer #3**

*The manuscript by Alizzi et al. describes the role of the RNA-binding protein Fne in dendrite morphogenesis of Drosophila da neurons. The authors identified defects in microtubule stabilization and dendrite-substrate attachment, which resulted in altered dendritic dynamics and reduced dendrite coverage. The authors then further searched for Fne target RNAs using the TRIBE technology and identified Bsg as one target that is responsible for at least partial function of Fne. Overall, the findings in this study suggest new mechanisms of how dendrite development is regulated by RNA-binding proteins and will be valuable to the field if the conclusions are well supported. However, I also have major concerns about the quality of some of the evidence for supporting their conclusions and the rigor of some their experiments. I think these issues need to be addressed before the manuscript can be considered for publication.*

*Major concerns:
1. A very important conclusion regarding the role of Fne is that it functions cell-autonomously to regulate neuronal cytoskeleton and dendrite-substrate interactions. However, in my opinion, this cell-autonomy has not been well established in the manuscript. I understand that a previous study suggests that Fne is specific to neurons based on in situ data and antibody staining. However, these expression analyses may not be sensitive enough to detect low expression in other tissues. In this regard, it is premature to imply that only neurons are affected in the whole animal mutant (e.g. referring to neurons in fne mutant as fne mutant neurons). As this is a very important foundation of this paper, can the authors show other evidence that Fne does not have a role in tissues like epidermal cells (e.g. RNAi, MARCM, etc).*

*Related to this point, does the image of Fne staining in Figure S1 include epidermal sections?*

- The immunofluorescence images in Fig. S1 do include epidermal sections, providing evidence that Fne is not expressed in the epidermis. More sensitive methods, RNA-Seq [FlyAtlas2] also found that Fne is exclusively expressed in the nervous system. Consistent with our identification of *fne* in a class IV da neuron RNAi screen, we show that two independent *UAS-fneRNAi* lines targeted specifically to class IV da neurons using *ppk-GAL4* recapitulate the empty space and dendrite crossing defects of *fne* mutants. Additionally, overexpression of *fne* specifically in class IV da neurons rescues the *fne* mutant defects, suggesting the defects seen in *fne* mutant neurons are the result of loss of *fne* in the neurons. Finally, to rule out a function for Fne in the epidermis, we used two different pan-epidermal drivers (*GAL4^69B^* and *GAL4^58A^*) to knock down *fne* and observed no defects. These two drivers have been used in many class IV da neuron papers to study the role of epidermal components in shaping class IV da neurons (Parrish et al. [2009] Neuron *63*, 788-802; Jiang et al. [2014] Development *141*, 2657-2668, Poe et al. (2017) PNAS *114*, E8062-E8071, Hoyer et al. [2018] Cell Rep. 24, 2261-2272 e5; Jiang et al. [2019] ELife *8*:e42455; Yang et al. [2019] Curr. Biol. *29*,1-15).

*2. Related to the first comment, Bsg::GFP expression levels/patterns on epidermal cells (outside dendrites) seem to be altered in the fne mutant (Figure 7). Can this be due to the epidermal function of Fne? Can the authors provide other evidence that neuronal Fne affects recruitment of epidermal Bsg::GFP to dendrites? A few ideas include overexpressing untagged Bsg::GFP in neurons, overexpressing Fne in neurons in the WT, and overexpressing Fne in the fne mutant.*

- We believe that differences in the epidermal levels of Bsg were due to slight differences in the age of the larvae. We have repeated the experiment with more careful staging of the larvae (Fig. 7A and B). Our immunofluorescence (Fig. S1) and RNAi analyses (Fig. S2) indicate that Fne expression is limited to class IV da neurons and it functions in a cell-autonomous manner.
- The experiments that the reviewer suggests would require expressing one transgene (*fne^OE^*) specifically in neurons, and expressing a different transgene (*UAS-GFP-Bsg*) in the epidermis. Expression these two transgenes simultaneously in different tissues would require combining the GAL4/UAS system with the Q system and to our knowledge there are no Q reagents available for expressing transgenes in class IV da neurons nor is there an appropriate Bsg transgene.

*3. The authors concluded that “Fne positively regulates Bsg levels in the neuron” (page 12). However, direct evidence is absent. Can Fne affect Bsg distribution instead of expression level? For example, in S2 cells expressing Fne, is Bsg mRNA level increased? I also don’t think the provided data convincingly show that Bsg tracks are derived from dendrites (Figure 6C”). In RNAi cells, there are still residual levels of Bsg::GFP in epidermal cells. The tracks could be epidermal Bsg proteins enriched at dendrite enclosure sites, like many other cell junctional proteins. A support for this argument is that Bsg::GFP tracks along dendrites are much brighter on the WT control epidermal cells.*

- We do not observe a difference in Bsg distribution. *bsg* mRNA levels do not increase in Fne-expressing S2 cells, nor did RNA levels for any of the Fne targets we tested, suggesting that Fne regulates translation or polyadenylation of its mRNA targets. Our point is that Bsg levels respond to Fne in a positive way, which could be accomplished through different modes of post-transcriptional regulation. Furthermore, our genetic analysis revealed that reducing levels of *bsg* suppressed the defects caused by *fne* overexpression (Fig. 7), indicating that a reduction in *bsg* expression level is important.
- We cannot rule out the possibility that there is some residual epidermal Bsg in the experiment shown in Fig. 6C”. However, the accompanying RNAi experiment, where we knockdown *bsg* in either the neuron or the epidermis (Fig. 6D-I), indicates that Bsg is expressed and required in both tissues. We attempted to knockdown *bsg* by RNAi in both neurons and epidermis of Bsg::GFP animals (i.e. performing the experiment in Fig. 6C with *ppk*-GAL4 driver added) but the combination of chromosomes required for this experiment proved to be lethal. We have modified the text to reflect the possibility that the tracks detected in Fig. 6C are due to residual epidermal Bsg and to indicate that the RNAi results provide evidence to the contrary.

*4. The results of Futsch staining shown in the manuscript are confusing and directly contradict many published results in the literature. For example, Futsch staining should be detected in dendrites of all da neurons, but Figures 3 and 7 suggest that it is specific to class IV da neurons. Second, the staining signal should not be limited to only the main trunks of primary dendrites but should also be in other stabilized dendrites even in the distal arbor. My assumption is that the limited distribution in neurons shown in these figures is due to low resolution and/or low detection power. Can the authors show higher-rez images with more sensitive detection?*

- These images have been masked to only show the signal in class IV da neurons. We apologize for omitting this critical information. We have added explanations to the figure legends.
- The distribution of Futsch signal that we observe closely mirrors that seen in previously published work (Stewart et al. [2012] J. Neurosci. *32*, 11631-42; Jenkins et al. [2017] *130*, 4120–4131; Hu et al. [2020] J. Neurosci. *40*, 1819-1833).
- Also see Sears and Brohier [2016] Dev Biol. *418*, 40–54: "in class IV neurons, Futsch is confined primarily to main branches (Grueber et al., 2002; Jinushi-Nakao et al., 2007)" and DeVault et al. [2018] Genes Dev. *32*, 402-414: "Previous studies have shown that Futsch is highly expressed in primary branches and at lower levels in secondary branches but is rarely detectable in tertiary branches (Ferreira et al. 2014; Sears and Broihier 2016)."

*5. I think the integrin expression data shown in Figure 4 are not very relevant. What does the change of integrin level/distribution in follicle cells tell us about the regulation in neurons? Also the results were not well described or well interpreted. Can the changes be simply in protein localization instead of expression (there seems to be more intracellular Mys in fne mutant). Are the changes in cell shape caused by changes of integrin?*

- These data have been moved to supplement, as per the suggestion of Reviewer 2. We quantified the total Mys intensity in individual cells, and the increase in this value upon *fne* overexpression cannot be attributed to a change in localization alone. The changes in cell shape are not caused by changes in integrin alone, and we do not claim that they are.

*6. The manuscript is generally lacking in statistical rigor, at least in the current form. First, the exact sample size for each experiment was not provided. In addition, some experiments had inadequate sample sizes (Figure 2D-E, Figure S2, and Figure 2H). In general, the sample size should be larger than 10. Lastly, wrong statistical methods were used for some experiments. For example, one-way ANOVA instead of t-test should be used when more than two groups are being compared (Figure 1E-1J, 2D-2E, 3D, 7G-7L), assuming that the data meet the criteria (normal distribution, equal variances, etc) for one-way ANOVA.*

- We have added the exact sample size to the figure legends. A sample size of 8-12 neurons for morphometric analysis is standard for the field (examples from 7 different labs: Sears and Brohier (2016) Dev. Biol. *418*, 40-54; Bhogal et al. [2016] Development 143, 2147–2159; Poe et al. [2017] PNAS *114*, E8062-E8071; Meltzer et al. (2017) Neuron *89*, 741-755; Im et al. (2018) Dis. Model Mech.*11*, dmm034231; Das et al. (2017) Genetics *207*, 1401-1421; Hoyer et al. [2018] Cell Rep. 24, 2261-2272e5. While the pupal quantifications have a sample size of 5, we feel the difficulty of the dissections and the strength of the data warrant the lower sample size.
- We have now performed ANOVA tests when warranted, and Kruskal-Wallis tests when the data do not meet the criteria for ANOVA tests. Thank you for pointing out this issue.

*7. I am not very sure if the real-time (should be called time-lapse, to be technically correct) imaging provided sufficiently convincing data for drawing a conclusion. First, the time duration seems a little too short to clearly visualize the growth dynamics. Second, the sample size is too low. Regarding the analysis, did the authors consider the change of dendrite length? Are the distances of dendrites to the soma considered, since fne LOF affects distal and proximal dendrites differently?*

- We have changed the wording to time-lapse. We and others have used similar durations in previously published work (Bhogal et al. [2016] Development 143, 2147–2159; Nagel et al. [2012] Development *16*, 2999-3009). We find that it is difficult to keep the animals alive and healthy for longer periods without specialized equipment and do not want to risk compromising the data by imaging for longer times when we can detect significant differences with the shorter time period. Our sample size is consistent with other published studies including Hu et al. [2020] J. Neurosci. *40*, 1819-1833, Nagel et al. [2012] Development *16*, 2999-3009, Bhogal et al. [2016] Development 143, 2147–2159) (3-4 neurons, with 20-35 branches/neuron). At each time point, we observed 1-5 dynamic branches in wild-type and 1-12 dynamic branches in *fne^–^* neurons, with the average being 2.5 for wild-type and 6.3 for *fne^–^* neurons.
- We redid the quantification to account for the distance of the branches from the soma. We have found that in *fne^–^* neurons, the increases in elimination and initiation exist in both proximal and distal regions of the arbor. For branch elimination, we observed a 1.6x increase compared to wild-type in the proximal region, and a 2.0x increase compared to wild-type in the distal region. For branch initiation, we observed a 2.1x increase in new branches compared to wild-type in the proximal region, and a 1.9x increase in new branches compared to wild-type in the distal region. These numbers are consistent with the ~2x increase in elimination and initiation that we observed in *fne^–^* neurons when distance from the soma was not considered. This suggests that a uniform branch instability exists throughout the entire arbor in *fne^–^* neurons. The empty space seen proximal to the soma in *fne^–^* neurons is the result of this instability, combined with the normal outward expansion of the neuron.

*Minor concerns:*

*1. Can the authors add some discussion to compare the roles of Fne and vertebrate HuD in dendrite morphogenesis?*

- We have added a comparison to the Discussion (p. 17).

*2. The fneOE data shown in Figure 1I and 1J seem inconsistent. fneOE has a larger critical radius than fne in 1J, but it seems to have a smaller critical radius in 1I.*

- Improved statistical analysis has helped to clear up this discrepancy. Our previous analysis using a t-test had incorrectly shown a significant difference in the critical radii of WT and *fne^OE^* neurons. Since there is no significant difference in the critical radii of the genotypes using ANOVA with Bonferroni-Holm *post hoc* test, we have removed this panel from the figure.

*3. The fne and fneOE phenotypes in pruning are very interesting, but the text has very little interpretation or discussion about these results. It would be good to discuss it in the discussion.*

- Thank you for pointing out this omission. We have added interpretation of these results to the Discussion (p. 15).

*4. There is very little explanation about the assay for detecting dendrite enclosure. I understand that the assay has been published. But some explanation is needed for readers to understand this experiment.*

- We apologize for the brevity. We have now added a comprehensive explanation to the Materials and Methods as well as information in the legends (p. 21).

*5. The descriptions for some results are vague. For example, Bsg is said to be expressed around the periphery of epidermal cells” (page 11). Does “periphery” mean the cell border, the plasma membrane, or something else?*

- We have changed the text to "cell borders" and have made additional wording changes to ensure clarity.

*6. Please indicate RNAi-expressing cells in hh-Gal4 RNAi images.*

- We did not include a marker for the *hh-GAL4* expressing cells in the experiments. However, we have cited Poe et al. [2017] PNAS *114*, E8062-E8071, who showed the domain of *hh-GAL4* expression in the epidermis. In Fig. 6B, C, this domain can be inferred from the abrupt boundary between the Bsg::GFP positive and negative epidermal cells.

*7. It’s not clear from the text, figure legends, or methods how some of the image panels were created (Figure 7A’, B’).*

- We have added more information to the figure legends to describe how the image panels were created.

*8. The data about the effects of bsg RNAi on dendrite crossing were not shown.*

- We have added these data in Fig. 6G.
